# Asparaginase combined with discontinuous dexamethasone improves antileukemic efficacy without increasing osteonecrosis in preclinical models

**DOI:** 10.1371/journal.pone.0216328

**Published:** 2019-05-06

**Authors:** Seth E. Karol, Laura J. Janke, John C. Panetta, Laura B. Ramsey, Xiangjun Cai, Monique A. Payton, David A. Jenkins, William E. Evans, Mary V. Relling

**Affiliations:** 1 Department of Oncology, St. Jude Children’s Research Hospital, Memphis, Tennessee, United States of America; 2 Department of Pathology, St. Jude Children’s Research Hospital, Memphis, Tennessee, United States of America; 3 Department of Pharmaceutical Sciences, St. Jude Children’s Research Hospital, Memphis, Tennessee, United States of America; European Institute of Oncology, ITALY

## Abstract

**Introduction:**

Combination therapy for acute lymphoblastic leukemia (ALL) is highly effective but results in significant toxicity including osteonecrosis. Asparaginase is known to potentiate both the antileukemic and osteonecrosis-inducing effects of dexamethasone. The schedule of dexamethasone alters osteonecrosis risk. However, the effects of the interaction with asparaginase are unknown when dexamethasone is given on a discontinuous schedule.

**Methods:**

Using the murine model of osteonecrosis, we compared the frequency of osteonecrosis in mice receiving discontinuous dexamethasone (3.5 days/ week) with mice receiving asparaginase and discontinuous dexamethasone. We then tested the effect on antileukemic efficacy using six pediatric ALL xenografts.

**Results:**

The addition of asparaginase to discontinuous dexamethasone did not alter the rate of osteonecrosis compared to dexamethasone alone (7/35 in dexamethasone with asparaginase combination vs. 10/36 in dexamethasone alone, p = 0.62) despite increasing steady-state plasma dexamethasone levels (103.9 nM vs. 33.4 nM, p = 9.2x10^-7^). Combination therapy with asparaginase and dexamethasone demonstrated synergistic antileukemic effects across all six xenografts studied.

**Conclusions:**

When discontinuous dexamethasone was given, its anti-leukemic activity synergized with asparaginase but the osteonecrosis-worsening effects of asparaginase (above dexamethasone alone) were not observed. Thus, there is a favorable drug interaction (unchanged toxicity, synergistic efficacy) between discontinuous dexamethasone and asparaginase.

## Introduction

Modern pediatric and pediatric-inspired regimens for the treatment of acute lymphoblastic leukemia (ALL) will cure 80–95% of children[1–5] and 60–83% of young adults.[6] Key differences between pediatric and adult regimens for ALL include the intensification of asparaginase and glucocorticoids such as dexamethasone in pediatric regimens, with the dose intensity of these agents partially credited for the improved outcomes in children.[6] When used together, these agents are known to increase the antileukemic efficacy of chemotherapy as well as the potential for the development of osteonecrosis.[7, 8]

Osteonecrosis (bone death) due to chemotherapy, can affect 15–45% of adolescents[9–12] and 8–17% of young adults[9, 12] receiving ALL therapy. Increased rates of osteonecrosis when dexamethasone and asparaginase are combined during post-remission therapy have led to a modification of dexamethasone dosing during these periods. The Children's Oncology Group (COG) trial CCG–1961 demonstrated a lower incidence of osteonecrosis when dexamethasone was given on an interrupted/ discontinuous schedule (dexamethasone given on days 1–7 and 15–21 instead of 1–21), even when two cycles of discontinuous therapy were compared to a single continuous cycle.[13] This result, combined with unacceptable rates of osteonecrosis when continuous dexamethasone was used, prompted changes from continuous to discontinuous dexamethasone schedules in recent trials.[14, 15] Two cycles of discontinuous therapy were also associated with improved antileukemic efficacy compared to a single continuous cycle.[13] However, it was unclear if the improved antileukemic effectiveness was due solely to dexamethasone or other chemotherapeutics employed during the second cycle.

To better understand these clinical outcomes, our group previously demonstrated discontinuous dexamethasone has equivalent antileukemic efficacy to continuous dexamethasone in both murine models of leukemia and patient derived xenografts of pediatric ALL.[16] We also demonstrated asparaginase added to continuous dexamethasone increases the rate of osteonecrosis in preclinical models.[17] However, it remains unclear whether asparaginase added to the less osteonecrotic[13, 18] and more clinically relevant discontinuous dexamethasone regimen will alter the rate of osteonecrosis. Here, we evaluate the addition of asparaginase to a discontinuous dexamethasone regimen and evaluate the effects on both osteonecrosis development and antileukemic efficacy using murine and human xenograft models, respectively.

## Materials and methods

All animal experiments were approved by the St. Jude animal use committee (protocol numbers 423–100428 for osteonecrosis experiments and 465–100549 for efficacy experiments). For osteonecrosis experiments, 4-week-old Balb/c male mice (locally bred, founding breeders from Jackson Laboratories, Ellsworth, Maine) were randomized to receive dexamethasone 4 mg/L (as dexamethasone sodium phosphate, American Pharmaceutical Partners, Inc., Schaumburg, IL) in the drinking water for 3.5 days/ week and either PEGylated asparaginase 1200 IU/kg IP (Oncaspar, Shire US Inc., Lexington, KY) or sham injection (normal saline, Hospira Inc., Lake Forest, IL) every 3.5 days. All mice received tetracycline (1 g/L continuously; Sigma-Aldrich, St. Louis, MO) and sulfamethoxazole/ trimethoprim (600/120 mg/L 3.5 days/ week; Aurobindo Pharma USA, Inc., Dayton, NJ) in the drinking water. Mice had free access to a folate deficient diet (Test Diet, Richmond, IN).[17, 18] Following 6 weeks of therapy, animals were humanely euthanized and osteonecrosis and epiphyseal arteriopathy were evaluated in the distal femur as previously described.[18, 19] Briefly, at the time of sacrifice, animals were humanely euthanized using carbon dioxide asphyxiation. Both femurs were collected, fixed in 10% formalin overnight, decalcified in Cal-Rite (Thermo Fisher Scientific, Waltham, MA), paraffin-embedded, sagittally sectioned, and stained with hematoxylin and eosin. Osteonecrosis was diagnosed in the distal femur if all the following were present: empty lacunae, ghost or pyknotic nuclei in osteocytes in the bone trabeculae, and necrosis of the adjacent marrow and stromal elements. Arteriopathy lesions were evaluated in arteriolar branches of the medial genicular artery on the surface of the distal femoral condyles.[19]

Xenografted patient-derived ALL cell lines were created as previously described.[16] Samples were selected based on *in vitro* sensitivity to chemotherapy measured by MTT as previously described.[20] For xenograft generation, frozen bone marrow aspirates obtained prior to therapy from children with newly diagnosed ALL were thawed, washed, and injected via tail vein in unirradiated 8-10-week-old female NOD Cg-Prdc^scid^ Il2rg^tm1Wjl^/SzJ (NSG) mice (Jackson Laboratories, Ellsworth, Maine). All patients/ families provided written informed assent/ consent for research studies including *in vitro* chemotherapy sensitivity and sample banking/ research use consistent with the Declaration of Helsinki. All research with human samples was approved by the St. Jude institutional review board (XPD10-102). Bone marrow from first-passage mice were transduced with lentiviral vector containing luciferase and YFP and reinjected for a second passage. Bone marrow from these mice was sorted and hCD45+/YFP+ cells were reinjected and expanded during passage three. Bone marrow from these animals were used for anti-leukemic efficacy experiments.

For efficacy experiments, unirradiated 7-9-week-old NSG female mice were injected with 1x10^6^ luciferase/YFP marked ALL cells obtained from passage 3 animals via tail vein. Treatment began 1–3 weeks after injection based on luciferase signal and the rapidity of engraftment during prior passages. Mice were randomized to receive no treatment, dexamethasone 8 mg/L in the drinking water for 3.5 days/ week, PEGylated asparaginase 200 IU/kg IP weekly, or both dexamethasone and asparaginase. Mice randomized to not receive asparaginase received sham injections of normal saline. All mice received antibiotics as in the osteonecrosis experiment. Mice had free access to standard chow (PMI 5013, Test Diet, Richmond, IN) and medicated water for the duration of treatment. Dosing of dexamethasone and asparaginase was based on prior data on dexamethasone pharmacokinetics [16, 21] and preliminary data on the pharmacokinetics and toxicity of asparaginase in these animals (not shown). Tolerated doses differ in the Balb/c mice needed for the osteonecrosis phenotype compared to the NSG mice needed for the ALL efficacy experiments.

The duration of treatment varied from 3–6 weeks based on the response of the treatment arms, with treatment ending when the luciferase signal in combination treated mice was undetectable above background. For the KMT2A sample (in which luciferase activity was lost), following 8 weeks of engraftment, mice received 2 weeks of treatment and were then humanely euthanized. Following euthanasia, bone marrow was evaluated using flow cytometry for leukemic involvement as measured by hCD45+ mononuclear cells.

Animals were monitored daily for evidence of toxicity (and in the case of leukemia bearing mice, leukemia progression) including changes in grooming, weight loss, decreased activity, or difficulty breathing. Leukemia bearing mice were also monitored with bioluminescence twice weekly, and in the case of T-cell leukemias and E2A–PBX1 leukemias, with peripheral blood flow cytometry weekly. Animals were humanely euthanized when moribund due to leukemia (as evidenced by hind limb paralysis, circling, or respiratory distress) or when leukemic burden reached a predetermined threshold (ventral luminescence reached 1x10^10^ p/s or peripheral blood flow cytometry of mononuclear cells contained greater than 50% hCD45+ cells). All blood collections and bioluminescent imaging was performed while animals were anesthetized with isoflurane.

The leukemic graft growth and effects of asparaginase and dexamethasone treatment were modeled using Gompertz growth and log kill as described in the following equation:

$$\frac{dy}{dt}=r∙\log\left(\frac{K}{y}\right)-(d_{1}∙ASP+d_{2}∙DEX+\alpha ∙ASP∙Dex)∙y;\;y(0)=y_{0}$$

where y is the leukemic burden (measured as ventral luminescence signal), y_0_ is the initial leukemic burden, r is the growth rate (increase in leukemia burden/day), K is the carrying capacity (leukemic burden units), d_1_ is the effect of asparaginase on the growth (1/day), and d_2_ is the effect of dexamethasone on the growth (1/day). The parameter α (1/day) is the interaction term for the combination of asparaginase and dexamethasone where α = 0 is an additive effect, α>0 is a synergistic effect, and α<0 is an antagonistic effect. ASP and DEX are step functions (0 or 1 if the drug is not present or present, respectively) describing when each drug is active. The model parameters were estimated using nonlinear mixed-effects modeling via Monolix (version 5.0.1). Luminescence data below the level of noise (3x10^6^) were censored using the censoring method available in Monolix.

To assess whether the dexamethasone and asparaginase demonstrated synergy in the KMT2A bone marrow, we used Bliss Additivity[22] to determine the change from additivity of the combination of dexamethasone and asparaginase. First the fraction of marrow leukemic involvement relative to the median no treatment result (Fu) was determined for dexamethasone alone, asparaginase alone, and the combination. The Bliss Additive effect was calculated as follows: Fu12 = Fu1Fu2 where Fu1 and Fu2 are the effects of dexamethasone alone and asparaginase alone, respectively and Fu12 is the Bliss additive effect of the combination. Synergy/ antagonism was determined by comparing the normalized effect of the combination effect of dexamethasone and asparaginase to the Bliss additive effect. If the combination effect was smaller than the Bliss additive effect then the interaction was considered synergistic. Likewise, if the combination effect was larger than the Bliss additive effect then the interaction was considered antagonistic.

Blood was obtained for pharmacokinetic analysis while mice were receiving therapy. Steady-state plasma for dexamethasone measurement was obtained after 3.5 consecutive days of treatment in all mice (just prior to beginning 3.5 dexamethasone-free days) and plasma for asparaginase activity was obtained 7 days after prior injections in leukemia bearing mice and 3.5 days after injection in osteonecrosis mice. Dexamethasone levels in plasma were determined using high-performance liquid chromatography with tandem mass spectrometry detection (HPLC-MS) with a linear reportable range of (5.1–1275 nM), as previously described.[16] Asparaginase activity was measured using a coupled enzymatic reaction as previously described, with a linear reportable range from 0.05–9.6 IU/ml.[23]

The frequency of osteonecrosis was compared using chi-square tests. Survival comparisons used log-rank testing. Comparison of population distributions of plasma chemotherapy levels and bone marrow involvement in the KMT2A mice utilized Wilcoxon rank sum testing. Statistical analyses were performed in R version 3.5.1.[24] Graphics were generated using R and GraphPad Prism version 7.00 for Windows (GraphPad Software, La Jolla California USA, www.graphpad.com). A significance threshold of p<0.05 was used for all analyses.

## Results

**Fig 1:**
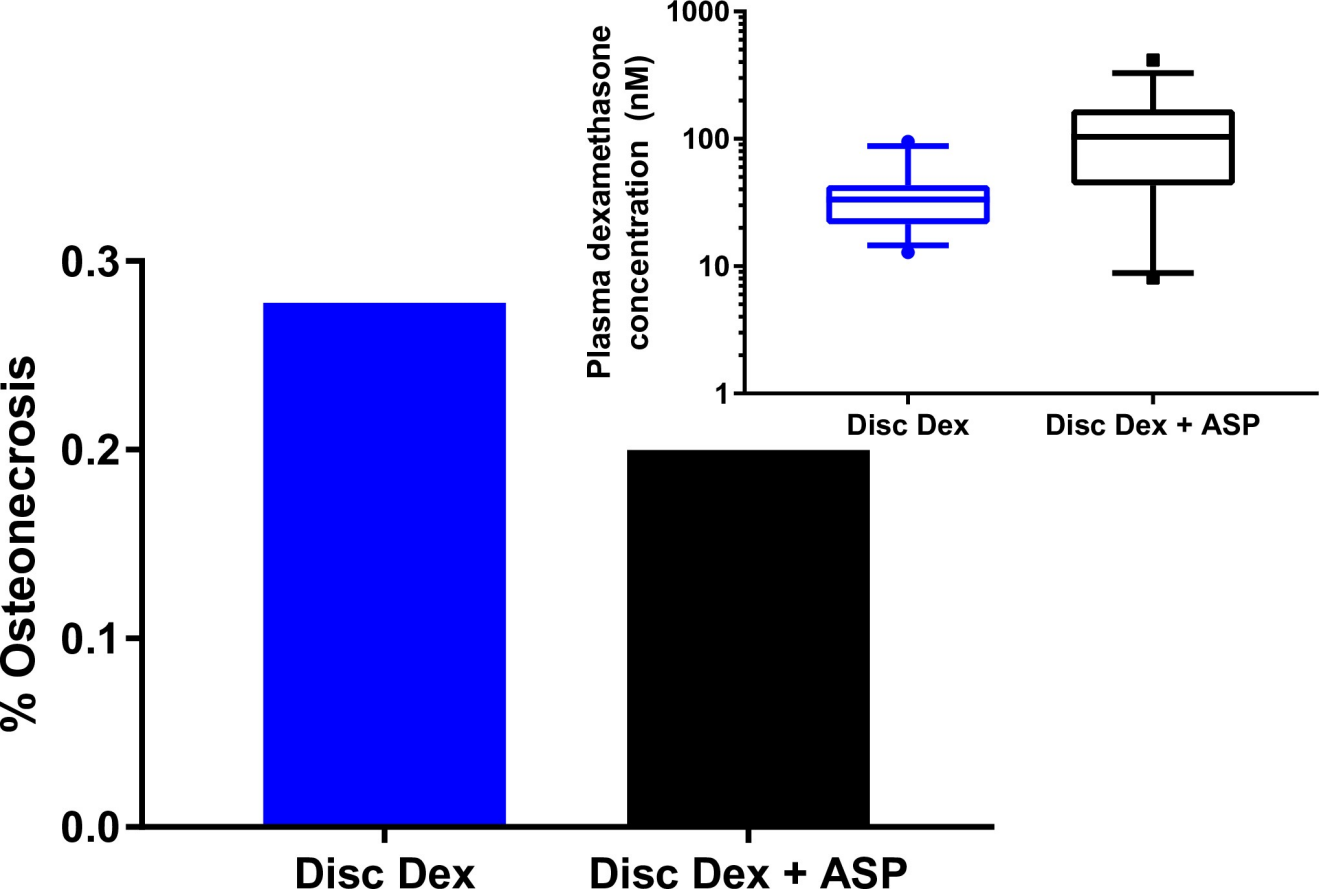
Osteonecrosis and pharmacokinetic results in mice receiving discontinuous dexamethasone and asparaginase. Rates of osteonecrosis were unchanged when asparaginase was added to discontinuous dexamethasone [7/35 (black) vs. 10/36 (blue), P = 0.62]. Plasma dexamethasone concentrations were higher in mice receiving asparaginase. Mice receiving both dexamethasone and asparaginase had plasma levels of dexamethasone 3-times higher than mice receiving dexamethasone alone (median 104nM vs. 33.4nM, p = 9.2x10^-7^, inset). Plotted values represent the median (center line of boxes), interquartile ranges (edges of boxes), median 80 percentiles (extended lines), and outlying values (dots/ squares).

The addition of PEGylated asparaginase to discontinuous dexamethasone did not increase the rate of osteonecrosis in mice (10/36 in dexamethasone alone vs. 7/35 in dexamethasone with asparaginase combination, p = 0.62, Fig 1). Extraosseous arteriopathy also did not differ in dexamethasone alone vs. dexamethasone with asparaginase groups (10/36 vs. 8/35 in combination group, p = 0.84). Median steady-state trough asparaginase activity in treated mice was 1.8 IU/ml [Inter-quartile range (IQR) 1.3–3.2], while activity was undetectable in all mice treated with dexamethasone alone. Dexamethasone levels at steady-state were higher in combination treated mice, with median levels of 103.9 nM (IQR 47.2–164.5 nM) vs. 33.4 nM (IQR 21.8–42.8 nM) in the dexamethasone monotherapy mice (p = 9.2x10^-7^, Fig 1 inset). Dexamethasone was undetectable in the plasma of all animals 3.5 days after stopping dexamethasone treatment.

**Fig 2:**
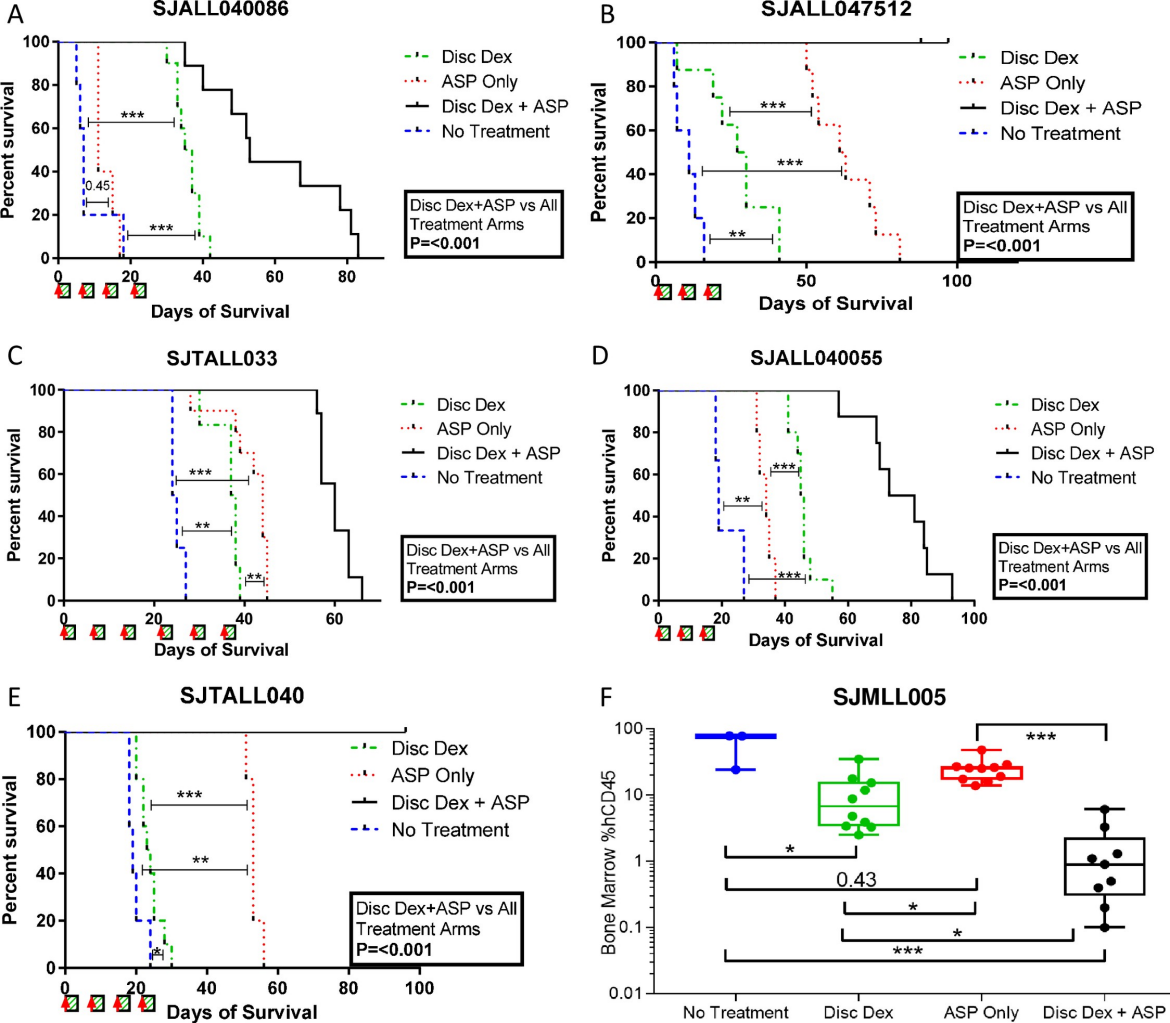
The combination of discontinuous dexamethasone and PEGylated asparaginase shows improved antileukemic efficacy in xenografts. Mice bearing ALL xenografts had prolonged survival (A-E) or lower bone marrow leukemic burden (F) with combination therapy (Disc Dex + ASP, black) compared to either asparaginase alone (ASP, red) or discontinuous dexamethasone alone (Disc Dex, green), all of which were superior to no treatment (blue). Red arrow: PEGylated asparaginase injection 200IU/kg intraperitoneal; Green box: dexamethasone (8 mg/L) in drinking water; *p<0.05; **p<0.01; ***p<0.001.

All five xenograft samples evaluated had prolonged leukemia-free survival with discontinuous dexamethasone compared to untreated mice (Fig 2). Four of the five had prolonged survival with asparaginase alone, with a slight increase in survival in the remaining sample (SJALL040086; Fig 2). The combination of dexamethasone and asparaginase was superior to either drug alone in all cases (p<0.001 for all samples). This improvement in therapy was evident in both leukemia-free survival (Fig 2) as well as in measures of synergistic interaction (Table 1, S1 Fig).

**Table 1:** Characteristics of patient derived xenografts at diagnosis and xenografted animals’ survival during anti-leukemic efficacy experiments.

Xenograft	Recurrent fusion	Pred LC50 at diagnosis	ASP LC50 at diagnosis	Survival days: Median (min-max)				Synergy coefficient (α), %CV
				Untreated	Disc Dex	ASP	Disc Dex + ASP	
SJTALL033	TCRB-LYL1	2.9nM*	<0.0016	24.5 (24–27)	37.5 (30–39)	44 (28–45)	60 (56–66)	0.58, 7%
SJTALL040	No recurrent fusions	10.5nM	0.0016	19 (18–24)	23.5 (20–30)	53 (51–56)	not reached	0.43, 10%
SJALL040055	TCF3-PBX1	7.5nM	0.0016	19 (18–27)	45.5 (41–55)	34 (31–37)	77 (57–93)	0.36, 8.7%
SJALL040086	ETV6-RUNX1	7.5nM	0.0016	7 (5–8)	36 (30–42)	11 (11–17)	53 (35–83)	0.34, 9.9%
SJALL047512	P2RY8-CRLF2	7.5nM	0.0016	11 (6–16)	28.5 (7–41)	62 (50–81)	not reached	0.61, 12%

For sample SJTALL033, the LC50 listed (*) is for dexamethasone, as no prednisone LC50 was available for this sample. The synergy coefficient for all samples was greater than 0, indicating a synergistic interaction. %CV: percent coefficient of variation, Disc Dex: discontinuous dexamethasone, ASP: PEGylated asparaginase, Disc Dex + ASP: discontinuous dexamethasone and PEGylated asparaginase.

**Fig 3:**
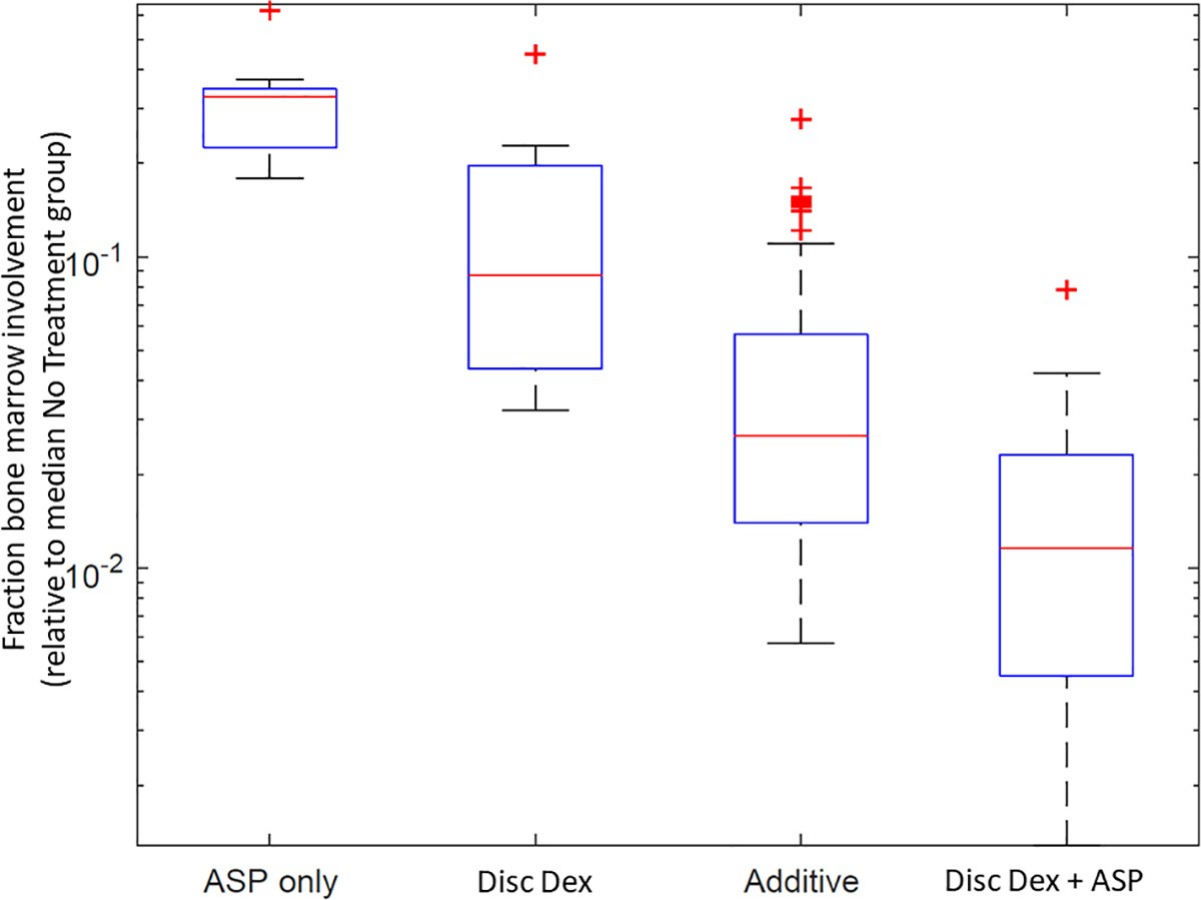
The combination of discontinuous dexamethasone and PEGylated asparaginase shows synergistic efficacy in bone marrow. Following two-weeks of treatment, mice engrafted with an KMT2A-rearranged leukemia and treated with either asparaginase alone (ASP only, left) or discontinuous dexamethasone alone (Disc Dex, left-center) both saw a reduction in bone marrow leukemic involvement as measured by flow cytometry compared to untreated mice (reference, not shown). Mice treated with the combination of discontinuous dexamethasone and asparaginase (Disc Dex + ASP, far right) had a greater decrease in marrow involvement than the additive effect (right-center) calculated by adding together the decrease seen from asparaginase alone and dexamethasone alone.

In one sample (KMT2A), leukemia progression was unable to be measured by either peripheral blood flow cytometry or luminescence. For this sample, bone marrow leukemic involvement was confirmed at the time of sacrifice by flow cytometry. Median marrow leukemic involvement was highest in untreated mice (77.8% of mononuclear cells, range 24.2–78.4%) and lowest in combination treated mice (0.9%, range 0.1–6.1%, p = 2.8x10^-8^ vs. untreated). Mice treated with either dexamethasone (median 6.8%, range 2.5–34.9%, p = 0.01 vs. untreated and p = 0.002 vs. combination) or asparaginase (median 25.5%, range 13.9–48.1%, p = 0.16 vs. untreated, p = 0.009 vs. combination, and p = 0.004 vs. dexamethasone) had intermediate levels of leukemic involvement, again demonstrating the improved therapeutic efficacy when both agents were combined (Fig 2F). This reduction in leukemic involvement in the combination treated mice (0.012) was greater than the calculated additive effect (0.027; p<0.01, Fig 3), indicating synergy. Leukemic involvement of the bones at the time of sacrifice precluded evaluation for osteonecrosis. [16] Moreover, it is unlikely that this strain [18, 25] or age [18] of mouse is susceptible to glucocorticoid-induced osteonecrosis, based on our extensive experience.[17–19, 25]

Pharmacokinetic sampling on leukemia bearing mice demonstrated both asparaginase and dexamethasone concentrations were within clinically relevant ranges. As in the osteonecrosis mice, dexamethasone concentrations were higher in mice receiving asparaginase (median 61.2 nM, IQR 32–81.5 nM) compared to mice receiving dexamethasone alone (median 19.2 nM, IQR 13.5–32.1, p = 8.5x10^-4^). Asparaginase activity levels were similar in mice receiving dexamethasone and asparaginase (median 0.36 IU/ml, IQR 0.25–0.61 IU/ml) compared to asparaginase alone (median 0.5 IU/ml, IQR 0.19–0.59 IU/ml, p = 0.97).

## Discussion

Discontinuous dexamethasone regimens have been widely adopted for use in post-remission therapy in pediatric and pediatric inspired ALL regimens.[2, 5, 13, 26–28] This change is based on both clinical and preclinical data suggesting that discontinuous dexamethasone regimens produce far less osteonecrosis than do regimens with 3-weeks of uninterrupted dexamethasone.[13–15, 18] We have previously demonstrated that discontinuous dexamethasone has equal antileukemic efficacy to uninterrupted dexamethasone in a variety of pediatric ALL xenografts.[16] However, dexamethasone is typically combined with asparaginase during delayed intensification, and a greater understanding of how this combination effects bony toxicity and antileukemic efficacy is needed to inform future preclinical and clinical studies.

Here, we demonstrated that the addition of PEGylated asparaginase to a discontinuous dexamethasone regimen which mirrors contemporary delayed intensification/ reinduction therapy does not increase the incidence of osteonecrosis in the murine model. This, combined with our prior work demonstrating the combination of asparaginase and continuous dexamethasone increases osteonecrosis,[17] suggests that the deleterious interaction between asparaginase and dexamethasone on the development of osteonecrosis is schedule dependent. Similar to findings in patients with ALL[8] and in murine experiments utilizing continuous dexamethasone,[17] the addition of asparaginase in this study increased the plasma levels of dexamethasone (Fig 1), presumably through the downregulation of hepatic metabolizing enzymes and transporters. However, despite this increased level of dexamethasone, asparaginase did not result in an increase in osteonecrosis above that observed with dexamethasone alone. The reason a discontinuous dexamethasone schedule is protective against osteonecrosis despite equivalent dexamethasone exposure to continuous regimens is unclear. The most likely reasons is that therapy-induced vascular endothelial injury[19] is less with discontinuous than continuous regimens, although the mechanism of this difference is unclear. Notably, our data indicates that asparaginase does not remove this protective effect despite increasing dexamethasone exposure. Although the weekly asparaginase regimen used in the current study resulted in lower steady-state trough levels than the twice-weekly regimen used with the continuous regimen (median 1.8 IU/ml vs. 5.4 IU/ml),[17] the decreased asparaginase levels are expected based on linear asparaginase pharmacokinetics, and both sets of levels are above trough levels observed in patients receiving ALL therapy.[29, 30] The weekly asparaginase paired with discontinuous dexamethasone better mimics the clinical schedules used than would a twice-weekly asparaginase schedule.[5, 13, 26] Thus, the lack of potentiation of osteonecrosis seen within the current study does not reflect differences in pharmacokinetics but rather differences due to changes in the underlying dexamethasone regimen. The use of concurrent enrollment in the arms with and without asparaginase also allowed for control of inter-experimental variability in the incidence of osteonecrosis in mice.[25]

Using ALL xenografts, we then demonstrated that the combination of discontinuous dexamethasone and asparaginase had synergistic antileukemic efficacy across a variety of ALL immunophenotypic and genetic subgroups. This suggests that the antileukemic benefits of combining these agents is broadly applicable in patients receiving therapy. While neither dexamethasone nor asparaginase was consistently superior to the other, both agents were consistently superior to no treatment and were consistently inferior to the combination regimen (Fig 2, Table 1). Although the xenografts treated here were sensitive to both dexamethasone and asparaginase *in vitro* (Table 1), all showed a synergistic benefit *in vivo* from combination therapy, suggesting that synergy occurs in samples which are sensitive to both agents. Moreover, although the *in vivo* single agent activity was moderate in some cases (i.e. SJALL040086 and SJMLL005 for asparaginase and SJTALL040 for dexamethasone), the combination therapy still showed significant synergy over the activity of the more active agent (e.g. for SJALL040086, although asparaginase alone did not significantly prolong survival, the combination therapy performed significantly better than dexamethasone alone) as shown in Table 1 and Figs 2 and 3. This suggests that the synergy observed still occurs even in ALL samples more resistant to either drug.

Together, our data indicate the feasibility of achieving synergistic antileukemic effects with asparaginase and dexamethasone without an undesired increase in osteonecrosis using simple changes to the schedule of combination chemotherapy. Our work demonstrates the need to consider schedule-dependent interactions between chemotherapeutic agents on the risk of both toxicity and anti-tumor efficacy when planning anti-cancer regimens.

## Supporting information

S1 Fig Ventral luminescence measurement of leukemic response to treatment. Ventral luminescence of diverse leukemic xenografts (panels A-E) demonstrate synergistic effects of discontinuous dexamethasone and asparaginase. Untreated mice (black) have rapid leukemic progression which is attenuated by both dexamethasone (blue) and asparaginase (green). The black horizontal line at 3 in the level of noise for the luminescence assay. For all samples, the decrease in ventral luminescence in the mice treated with both discontinuous dexamethasone and asparaginase (red) is greater than the calculated additive decrease (magenta), indicating synergy, which is quantified above each plot and summarized in Table 1.
(PDF)
