# Supplementary material for: Asparaginase combined with discontinuous dexamethasone improves antileukemic efficacy without increasing osteonecrosis in preclinical models
Source: PLoS One. 2019 May 6;14(5):e0216328. doi: 10.1371/journal.pone.0216328 (PMC6502315; doi:10.1371/journal.pone.0216328)
Supplement: S1 Fig — Ventral luminescence of diverse leukemic xenografts (panels A-E) demonstrate synergistic effects of discontinuous dexamethasone and asparaginase. Untreated mice (black) have rapid leukemic progression which is attenuated by both dexamethasone (blue) and asparaginase (green). The black horizontal line at 3 in the level of noise for the luminescence assay. For all samples, the decrease in ventral luminescence in the mice treated with both discontinuous dexamethasone and asparaginase (red) is greater than the calculated additive decrease (magenta), indicating synergy, which is quantified above each plot and summarized in Table 1. (PDF) [file pone.0216328.s001.pdf]

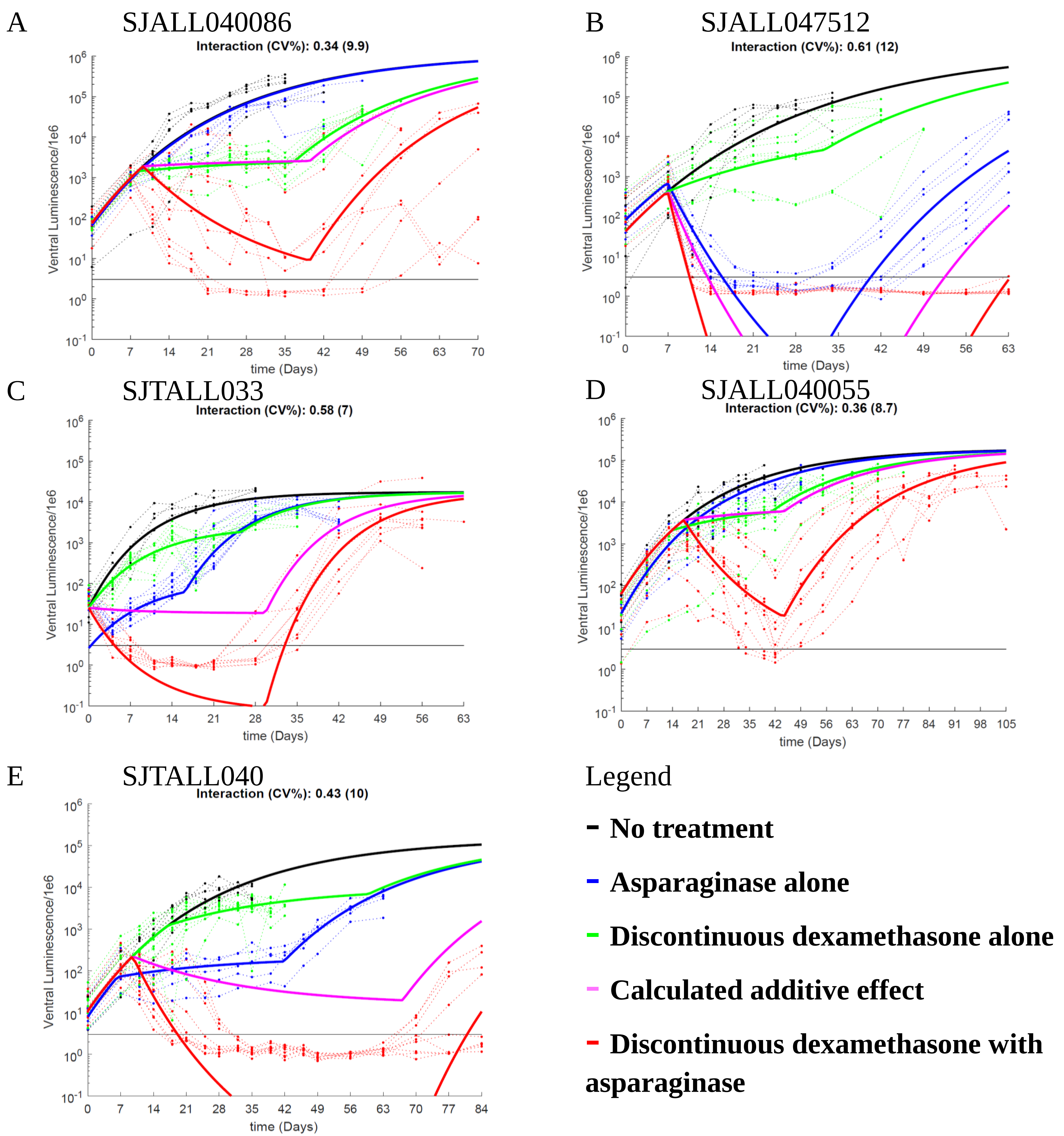
A
SJALL040086
Interaction (CV%): 0.34 (9.9)
B
SJALL047512
Interaction (CV%): 0.61 (12)
C
SJTALL033
Interaction (CV%): 0.58 (7)
D
SJALL040055
Interaction (CV%): 0.36 (8.7)
E
SJTALL040
Interaction (CV%): 0.43 (10)
Ventral Luminescence/1e6
time (Days)
Legend
No treatment
Asparaginase alone
Discontinuous dexamethasone alone
Calculated additive effect
Discontinuous dexamethasone with asparaginase
